# *MtGA2ox10* encoding C20-GA2-oxidase regulates rhizobial infection and nodule development in *Medicago truncatula*

**DOI:** 10.1038/s41598-019-42407-3

**Published:** 2019-04-11

**Authors:** Goon-Bo Kim, Seong-Uk Son, Hee-Ju Yu, Jeong-Hwan Mun

**Affiliations:** 1 Department of Bioscience and Bioinformatics, Myongji University, Yongin, 17058 Korea; 2 Department of Life Science, The Catholic University of Korea, Bucheon, 14662 Korea

## Abstract

Gibberellin (GA) plays a controversial role in the legume-rhizobium symbiosis. Recent studies have shown that the GA level in legumes must be precisely controlled for successful rhizobial infection and nodule organogenesis. However, regulation of the GA level via catabolism in legume roots has not been reported to date. Here, we investigate a novel GA inactivating C20-GA2-oxidase gene *MtGA2ox10* in *Medicago truncatula*. RNA sequencing analysis and quantitative polymerase chain reaction revealed that *MtGA2ox10* was induced as early as 6 h post-inoculation (hpi) of rhizobia and reached peak transcript abundance at 12 hpi. Promoter::β-glucuronidase fusion showed that the promoter activity was localized in the root infection/differentiation zone during the early stage of rhizobial infection and in the vascular bundle of the mature nodule. The CRISPR/Cas9-mediated deletion mutation of *MtGA2ox10* suppressed infection thread formation, which resulted in reduced development and retarded growth of nodules on the *Agrobacterium rhizogenes*-transformed roots. Over-expression of *MtGA2ox10* in the stable transgenic plants caused dwarfism, which was rescued by GA_3_ application, and increased infection thread formation but inhibition of nodule development. We conclude that *MtGA2ox10* plays an important role in the rhizobial infection and the development of root nodules through fine catabolic tuning of GA in *M. truncatula*.

## Introduction

Nodulation is the mutual interaction between legume plants and rhizobial bacteria that forms a symbiotic nitrogen-fixing nodule. The process is tightly controlled by the host plant via the nodulation signaling pathway, wherein plant hormones including cytokinin, auxin, ethylene, and gibberellin (GA) participate (reviewed by Oldroyd^1^). The roles of GA in nodulation of legume species are controversial and both positive and negative effects have been reported. Pea *na*, a loss-of-function mutant of the *ent*-kaurenoic acid oxidase gene (*KAO*), was characterized by a reduction in the size and number of nodules, indicating that GA is required to support nodule formation^2^. In contrast, other studies have indicated negative roles of GA in nodulation. In *Lotus japonicus* and *Medicago truncatula*, exogenous GA application at concentration ranges of 0.1 to 1 µM resulted in inhibition of rhizobial infection and nodule organogenesis^3,4^. Considering the fact that root hair deformation was also reduced by GA application, the negative effect of GA on nodulation was proposed to act at the very early stage of the Nod factor signaling^3^. Negative regulation of the number of nodules formed by exogenous GA was shown to be mediated by the DELLA protein, which can interact with NSP2 and NF-YA1 *in vitro*^4^. Over-expression of *MtDELLA1* increased infection thread formation without changes in nodule number. However, null mutant *della* or RNAi knockdown plants had reduced numbers of infection thread and nodule formation^2,4,5^. Nodules formed in the *della* lines were similar in appearance to those of the wild types and still fixed the same amount of N as the wild types in pea. In addition, GA-deficient mutant plants recovered normal nodule organogenesis via knockout of DELLA^5^. Based on these results, a dual role of GA in two distinct stages of nodule organogenesis was proposed; the suppression of infection thread formation and promotion of nodule development^6^. A recent study validated this hypothesis by using various mutant pea plants with defective GA biosynthesis or signaling pathways^5^.

**Figure 1:**
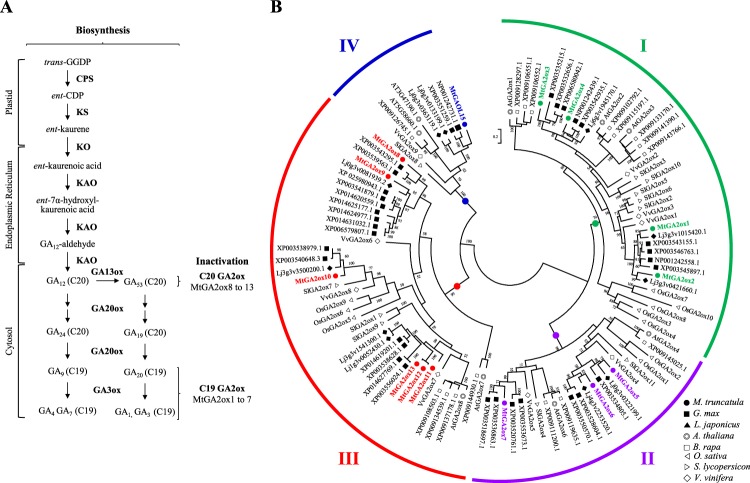
Schematic representation of gibberellin biosynthesis pathway and *GA2-oxidase* gene family in plant. (**A**) The major GA biosynthesis and metabolic pathway showing intermediate molecules, final products, and responsible enzymes for every step. GGDP, geranylgeranyl diphosphate; CPS, *ent*-copalyl diphosphate synthase; CDP, *ent*-copalyl diphosphate; KS, *ent*-kaurene synthase; KO, *ent*-kaurene oxidase; KAO, *ent*-kaurenoic acid oxidase; GA13ox, GA13-oxidase; GA20ox, GA20-oxidase; GA3ox, GA3-oxidase; C20 GA2ox, C20-GA2-oxidase; C19 GA2ox, C19-GA2-oxidase. (**B**) Phylogenetic relationship of the plant *GA2-oxidase* gene family. A Maximum-Likelihood tree was constructed using the deduced amino acid sequences of 9 *A. thaliana*, 16 *B. rapa*, 33 *G. max*, 11 *L. japonicus*, 14 *M. truncatula*, 11 *S. lycopersicon*, and 9 *V. vinifera* putative *GA2-oxidase* genes. Groups of genes are represented by color arcs. Bootstrap values are indicated on the branches and the branch length reflects the substitution per site. Groups I to III of *GA2ox* are designated according to the criteria suggested for *A. thaliana* and *S. lycopersicon*^31^. Group IV is comprised of dual specificity *GA2-oxidase like* (*GAOL*) genes. Closed symbols denote legume species (*G. max*, *L. japonicus*, and *M. truncatula*) and open symbols denote non-legume species. Genes of *M. truncatula* are color coded according to their group.

In higher plants, biosynthesis of GA occurs first in the plastid where *trans*-geranylgeranyl diphosphate is converted to *ent-*copalyl diphosphate and then to *ent*-kaurene by serial action of *ent-*copalyl diphosphate synthase (CPS) with *ent-*kaurene synthase (KS). A tetracyclic diterpene *ent*-kaurene is oxidized to *ent*-kaurenoic acid by *ent*-kaurene oxidase (KO) and further converted to GA_12_ by KAO on the membrane of the endoplasmic reticulum. GA_12_ can be oxidized to GA_53_ by GA13-oxidase (GA13ox). In the cytosol, GA_12_ and GA_53_ are further oxidized to bioactive GAs through the early 13-hydroxylation pathway or the non-hydroxylation pathway by a series action of GA20-oxidase (GA20ox) and GA3-oxidase (GA3ox). At each step, intermediate or bioactive GAs can be oxidized by GA2-oxidase (GA2ox), leading to the inactivation of these hormone molecules^7^. There are two types of GA2ox in the catabolic pathway for GAs^8^. Initially identified GA2ox utilized bioactive C19 GAs (GA_1_ and GA_4_) and their immediate precursor (GA_20_ and GA_9_) as substrates. Later, a novel type of GA2ox was discovered, which contained three unique conserved amino acid motifs and catalyzed only earlier intermediate C20 GAs (GA_12_ and GA_53_) (Fig. 1A). The ‘Janus face’ of GA on nodulation^9^ suggested that GA biosynthesis and inactivation must be precisely regulated in accordance with the progress of nodule organogenesis. Therefore, root GA concentration should be maintained at a low level at the early stage of epidermal rhizobial infection and then at a high level at the later stage of nodule organogenesis.

The cellular level of bioactive GA can be regulated in several ways, including transport of precursors or active forms of GA into the cells, inactivation of bioactive GA, or transcriptional regulation of genes involved in the biosynthesis and catabolic pathways (reviewed by Olszewski *et al*.^10^). As demonstrated in the reproductive transition of rice^11^ and *Lolium*^12^, regulation of GA transport via the vascular system is responsible for controlled organ development. GA_12_, the first GA compound produced by the GA biosynthesis pathway, is imported into the cytosol; it is then further oxidized by GA oxidases and converted to the bioactive form of GAs^10^. Recently, GA_12_ was identified as the major form of GA responsible for long-distance transport through the vascular system^13,14^. This finding is consistent with the expectation that GAs involved in long-distance transport should be inactive to avoid any nonspecific effects, and then converted to an active form at the location where the active GAs are required. The GA-deficient pea mutant *na* had dwarfism and decreased nodule formation due to disruption of production in GA_12_ precursor that ultimately leads to reduction in bioactive GA_1_^15^. Therefore, control of GA_12_ metabolism is expected to be an effective means to regulate the pools of precursors of downstream GA biosynthesis. The cellular GA level can also be changed through inactivation of the bioactive forms by GA2ox^13^. The major GA inactivation enzyme is C19 GA2ox^16^ and the significance of C20 GA2ox was demonstrated by floral initiation in *Arabidopsis thaliana*^17^. Over the last decade, transcriptional regulation of genes related to the GA biosynthesis pathway in legume plants has been investigated, which has provided a comprehensive understanding of the dynamic nature of GA regulation. Gene expression studies revealed that the GA biosynthetic pathway genes are regulated in response to rhizobial inoculation or Nod factor treatment. For example, *SrGA20ox1* of *Sesbania rostrata* was upregulated during lateral root-based nodulation and its infection-related expression pattern was dependent on Nod factors^18^. Similarly, several *GA20ox* and *GA3ox* genes of soybean were upregulated during the early stage of nodulation at 12 and 48 h after rhizobial inoculation^19,20^. Early GA precursor biosynthesis genes were also highly expressed upon rhizobium inoculation of the root hair cells of *M. truncatula*^21^.

Most of our current understanding of the roles of GA in symbiotic nodulation is based on mutant or gene studies of GA biosynthesis-related genes in pea and *DELLA* in *L. japonicus* and *M. truncatula*^2–5,15,22,23^. However, genes related to inactivation or catabolic regulation of GA during symbiotic nodulation of legume plants have not been studied to date. Previously, we investigated massive temporal transcriptome dynamics of nodulation signaling in *M. truncatula* wild-type cv. Jemalong A17, compared to mutants with absent (*nfp*^24^) or decreased Nod factor sensitivity (*lyk3*^25^) and an ethylene-insensitive mutant (*skl*^26^) at the early symbiotic stages (0 to 48 h post-inoculation [hpi]) with rhizobia^27^. Among the thousands of novel candidate genes undergoing Nod factor-dependent and ethylene-regulated expression, GA biosynthesis and signaling pathway genes were enriched at 12 hpi when root hair deformation and branching occurred. We surveyed the GA-related genes in a list of symbiosis-specific genes in which transcription was activated by Nod factors, and found a partial complementary DNA (cDNA) sequence showing similarity to *GA2ox* that mapped to the Medtr4g074130 locus in the recent *M. truncatula* genome release (Mt4.0).

In this study, we first report the functional characterization of *MtGA2ox10* encoding a novel C20 GA catabolic enzyme in symbiotic nodulation. We combine phylogenetic sequence comparison, expression analyses using RNA sequencing (RNA-seq) data and quantitative polymerase chain reaction (qPCR), native promoter::β-glucuronidase (GUS) fusion, CRISPR/Cas9-mediated gene deletion, and over-expression experiments. Our findings suggest that *MtGA2ox10* plays important roles in both rhizobial infection at an early stage and nodule development at a late stage of symbiotic nodulation in *M. truncatula*.

## Results

### Genome-wide identification of *GA2ox* genes in *M. truncatula*

The *MtGA2ox* genes were identified based on a BLASTP search of all *M. truncatula* reference gene models against the *A. thaliana GA2ox* gene family, including seven *GA2ox* genes and two *GA2-oxidase like* (*GAOL*) genes, defined in the METACyc database^28^. A total of 13 *MtGA2ox* genes and 1 *MtGAOL* gene were identified from the *M. truncatula* genome (Mt4.0) and were named as *MtGA2ox1*-*13* and *MtGAOL15* (Fig. 1A and Supplementary Table S1). None of the *M. truncatula* orthologs to *AtGA2ox3*, *AtGA2ox7*, and *AT3G47190.1* were identified, whereas C20 GA-specific *GA2ox* genes in *M. truncatula* were present and outnumbered *A. thaliana* by six genes (*MtGA2ox8* to *13*) to one gene (*AtGA2ox8*).

The phylogenetic relationship of the *MtGA2ox* gene family with its homologs in the sequenced plant genomes was reconstructed to investigate and characterize the phylogenetic patterns of the subgroups (Fig. 1B). A total of 113 deduced amino acid sequences of *GA2ox* and *GAOL* identified from eight sequenced plant genomes, including *A. thaliana*, *Brassica rapa*, *Glycine max*, *L. japonicus*, *M. truncatula*, *Oryza sativa*, *Solanum lycopersicon*, and *Vitis vinifera*, were multiple aligned to construct a phylogenetic tree. A Maximum-Likelihood tree using the protein sequences of the *GA2ox* genes showed that the plant *GA2ox* gene family is divided into four major clades. Groups I to III consist of *GA2ox* and Group IV includes only *GAOL*. Interestingly, Group I and II contain C19 GA-specific *GA2ox* (C19 *GA2ox*), whereas Group III comprises C20 GA-specific *GA2ox* (C20 *GA2ox*). Moreover, Group III *GA2ox* genes contained three unique conserved amino acid motifs that are absent in C19 *GA2ox* (Supplementary Fig. S1) and were relatively abundant in legume species (4–15 genes) compared to the non-legume species (1–4 genes). In each Group, legume (*G. max*, *L. japonicus*, and *M. truncatula*) and crucifer (*A. thaliana* and *B. rapa*) genes clustered into taxa-specific subgroups, indicating the close evolutionary relationship of genes in the same family.

### *MtGA2ox10* is the unique gene of the *MtGA2ox* gene family induced by rhizobium inoculation

**Figure 2:**
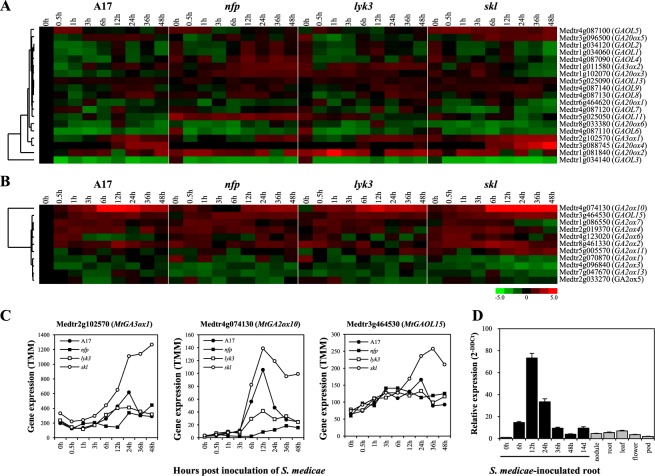
Expression of gibberellin (GA) biosynthesis and inactivation pathway genes in *M. truncatula* at various times after *S. medicae* inoculation. (**A,B**) Heatmaps representing the expression of GA biosynthesis (**A**) and inactivation (**B**) pathway genes in A17, *nfp*, *lyk3*, and *skl* based on relative log-transformed expression values (average trimmed mean of M-values [TMM] counts normalized to A17 at 0 hpi) of genes that were quantified from RNA sequencing data (NCBI BioProject accession No. PRJNA269201). The vertical axis dendrogram organizes genes according to their coexpression. The horizontal axis shows the genotype-specific time course (0–48 hpi with rhizobium). (**C**) Line graphs showing average TMM counts normalized to A17 at 0 hpi for *MtGA3ox1*, *MtGA2ox10*, and *MtGAOL15*. (**D**) Expression of *MtGA2ox10* in various tissues verified using quantitative polymerase chain reaction. Values (the comparative cycle threshold [2^−DDct^]) represent the relative expression calculated using the 0 hpi sample as a reference. Error bars depict the standard error calculated from three independent biological replicates.

To examine the expressional characteristics of each *MtGA2ox* gene as well as other genes related to GA biosynthesis in response to rhizobial infection, we investigated the expression pattern of the genes by searching the *Medicago truncatula* Gene Expression Atlas (MtGEA^29^) database, and by transcriptome analysis based on our large scale RNA-seq data from A17, *nfp*, *lyk3*, and *skl* roots that were inoculated with *Sinorhizobium medicae* ABS7M^27^. In MtGEA, none of the genes related to GA biosynthesis and inactivation exhibited nodule-specific expression (data not shown). In the transcriptome analysis using RNA-seq data, 19 out of 22 GA biosynthesis-related genes (6 *GA20ox*, 2 *GA3ox*, and 14 *GAOL*) and 11 out of 14 *GA2ox* genes were expressed in *M. truncatula* roots (Supplementary Table S2). Among these genes, one GA biosynthesis-related gene (*MtGA3ox1*) and two GA inactivation-related genes (*MtGA2ox10* and *MtGAOL15*) showed transcriptional changes between the genotypes, which occurred between several hours to 2 days post-inoculation (dpi) with *S. medicae* (Fig. 2A,B). Their transcriptions responded to rhizobium inoculation in the wild type at 12 or 24 hpi and were markedly enhanced in *skl* (Fig. 2C). Of particular interest was that *MtGA2ox10* was transcriptionally up-regulated at 6 hpi, peaked at 12 hpi where its expression level was approximately 3- to 5-fold higher than that in *nfp* and *lyk3*, and slowly declined over the rest of the time course. In contrast, *MtGA3ox1* and *MtGAOL15* showed similar expression patterns in A17, *nfp*, and *lyk3* over the time course. The peak expression of these genes in A17 at 24 hpi was only 1.4- to 1.5-fold higher than those in *nfp* and *lyk3* (Fig. 2C). Therefore, *MtGA2ox10* was a unique member of the GA metabolic pathway genes in *M. truncatula*, which showed up-regulation in a rhizobia-dependent and ethylene-regulated manner between 6 and 48 hpi. Moreover, the rhizobia-dependent induction of *MtGA2ox10* required *NFP* and *LYK3*, indicating that its transcription occurs downstream of Nod-factor recognition.

Transcriptional induction of *MtGA2ox10* in *M. truncatula* root by rhizobium inoculation was evaluated by qPCR analysis of gene expression in a series of root samples and in different tissues, namely nodules, leaves, flowers, and pods (Fig. 2D). Consistent with the results of the RNA-seq data analysis, expression of *MtGA2ox10* was highly specific to the early stage of *S. medicae* inoculation. Transcription of *MtGA2ox10* was barely detected from the un-inoculated roots at 0 hpi. However, the transcript level increased from 6 hpi, peaked at 12 hpi with a ~73-fold increase in transcript abundance compared to 0 hpi, and then gradually declined until 48 hpi. Expression of *MtGA2ox10* was also detected in the mature nodule and other tissues, including roots in the absence of rhizobia, leaves, flowers, and pods; however, the levels were lower than those of the rhizobium-inoculated roots at 36 hpi.

### *MtGA2ox10* is expressed in symbiotic tissues and nodules

Transcriptional fusion of the native *MtGA2ox10* promoter and *GUS* reporter gene was used to examine temporal and spatial patterns of expression in transformed hairy roots of wild type A17 plants. The *MtGA2ox10pro::GUS* fusion construct exhibited an expression pattern nearly identical to that in the qPCR experiment, with GUS activity detected from 1 hpi, peaking at 12 hpi and declining thereafter (Supplementary Fig. S2). To characterize the tissue-level activation of the *MtGA2ox10* promoter in roots and nodules, the distribution of GUS activity in symbiotic tissues was assessed by histochemical staining and microscopic analyses of the specimens.

**Figure 3:**
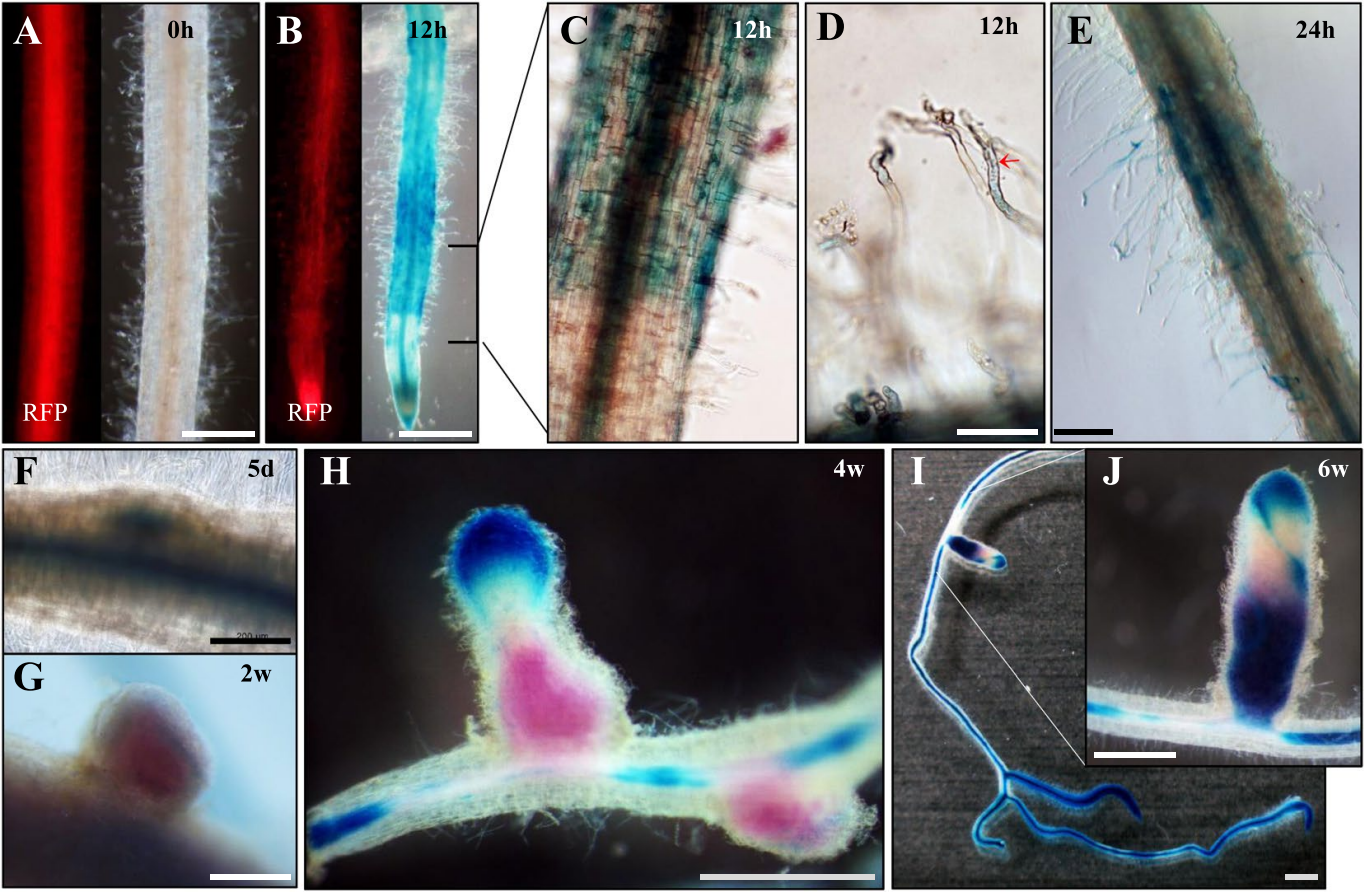
Histochemical localization of *MtGA2ox10* promoter activity in *M. truncatula* roots and nodules. (**A–J**) *M. truncatula* roots transformed using the *MtGA2ox10pro::GUS* construct and stained using X-Gluc as a substrate. (**A**) Uninoculated roots expressing red fluorescent protein (RFP) at 0 hpi. (**B,C**) Roots at 12 hpi. (**D**) Deformed root hairs at 12 hpi. Arrow denotes infection thread. (**E**) Roots at 24 hpi. (**F–J**) Nodules at different stages of development (5 dpi to 6 wpi). (**G–J**) *S. medicae* expressing a *LacZ* construct stained using Magenta-Gal as a substrate. Scale bars are 1 mm (**A,B,H–J**) or 200 µm (**D–G**).

In the absence of rhizobium inoculation, *MtGA2ox10pro::GUS* expression was not detected in roots (Fig. 3A). Inoculation of transgenic roots with *S. medicae* induced strong expression of *MtGA2ox10pro::GUS* at 12 hpi, with GUS activity differing between different zones; GUS activity was detected in the entire root area (epidermis, cortex, and vascular tissues), in the differentiation or maturation zone, in the vascular tissues in the elongation zone, and in the apical meristem and apices of the root cap (Fig. 3B). Interestingly, only infected or deformed root hairs in the differentiation zone exhibited GUS staining (Fig. 3C). GUS activity was reduced but localized to infected root hairs and cortex tissues, where infection thread extended at 24 hpi (Fig. 3E). At 5 dpi, strong expression of the *MtGA2ox10* promoter was detected in both nascent nodules and vascular tissues (Fig. 3F). *MtGA2ox10pro::GUS* expression in functional nodules was observed throughout the outer layers of developing nodules at 2 wpi, and in the meristem and infection zone of mature nodules at 4 wpi, without any overlapping Magenta-Gal-stained bacterial *LacZ* expression in the nitrogen fixation zone (Fig. 3G,H). Root vascular bundles at 4 wpi also showed GUS activity, except in the regions basal to the nodules. Similar expression patterns persisted at 6 wpi, while GUS staining was also detected in the nitrogen fixation and senescent zones of the nodule (Fig. 3I,J).

### Deletion mutation of *MtGA2ox10* reduces nodule number and retards nodule development

**Figure 4:**
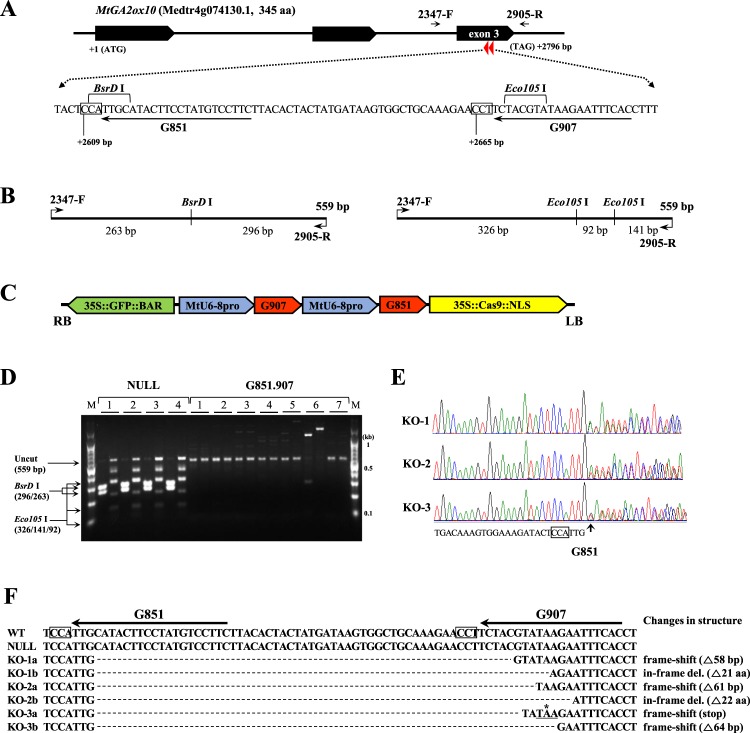
CRISPR/Cas9-mediated deletion mutation of *MtGA2ox10*. (**A**) Gene structure of *MtGA2ox10*. Target sequences of two guide RNAs, G851 and G907, were designed on exon 3. Relative nucleotide positions of the PAM sites marked in boxes are numbered from the start codon. Restriction sites for *BsrD* I and *Eco105* I are also presented. (**B**) Restriction maps of the wild type PCR products amplified with the 2347-F and 2905-R primers for genotyping by restriction fragment length polymorphism (RFLP), using *BsrD* I (left) or *Eco105* I (right). (**C**) T-DNA structure of the Cas9 binary construct G851.907 for the deletion mutation of *MtGA2ox10*. Two single guide RNAs (sgRNAs) (G907 and G851 under *MtU6–8* promoters) were tandem-assembled into the binary pGK3304 vector, which contains the *GFP::BAR* selection marker and *Cas9::NLS* under *CaMV 35S* promoters. (**D**) PCR-RFLP genotyping of *A. rhizogenes*-transformed roots harboring G851.907. PCR amplicons from four root samples of the pGK3304 empty vector (NULL) and seven root samples of G851.907 (G851.907) were digested independently by *BsrD* I (left lane) or *Eco105* I (right lane). Note that all the amplicons from the NULL samples were digested to fragments of the expected sizes, as shown in the left margin of the agarose gel. In contrast, none of the amplicons from G851.907 samples were digested, indicating disruption of the restriction sites for *BsrD* I and *Eco105* I. Sample 6 shows an increased amplicon size, presumably due to an insertion. (**E**) Sanger sequencing chromatograms for the PCR amplicons of the G851.907 KO-1, -2, and -3. The PAM sequence for G851 is denoted in the box and the expected cleavage site (−4 bp from PAM) is marked with an arrow, where mixed peaks appear in the sequencing chromatograms. (**F**) Sequences of each allele in the G851.907 KO-1, -2 and -3. The PAM sequences for sgRNAs are shown in boxes. Predicted changes in the protein structure by deletion mutations are described on the right.

For the loss-of-function analysis of *MtGA2ox10*, CRISPR/Cas9 was utilized to generate a deletion mutation of *MtGA2ox10* in *Agrobacterium rhizogenes*-mediated transformed roots. We used the promoter of the *M. truncatula U6–8* small nuclear RNA gene^30^ instead of *A. thaliana U6–26* for efficient transcription of guide RNAs in the transformed roots of *M. truncatula*. To introduce a large deletion in motif 6 of the GA2ox family which functions as an oxygenase^31^, co-expression of two distinct guide RNAs was carried out; two single guide RNAs (sgRNAs; G851 and G907) were designed on exon 3 of *MtGA2ox10* (Fig. 4A,B) and placed together in a single vector under the control of the *MtU6–8* promoter, resulting in a dual sgRNA construct (G851.907; Fig. 4C). Screening of transformed roots by PCR-restriction fragment length polymorphism (RFLP) with *BsrD* I and *Eco105* I, as well as PCR amplicon sequencing, revealed that 19% (7 out of 36) of the transformed roots expressing green fluorescent protein (GFP) harbored deletion mutations in the target region (Fig. 4D,E and Supplementary Fig. S3). Among the transformed roots with edited *MtGA2ox10*, three samples (G851.907 KO-1 to 3) were selected and further analyzed. G851.907 KOs were characterized by heterozygous biallelic sequences with large deletions between the G851 and G907 target regions, resulting in an in-frame deletion, frame shift, or premature stop codon in the reading frame (Fig. 4F).

**Figure 5:**
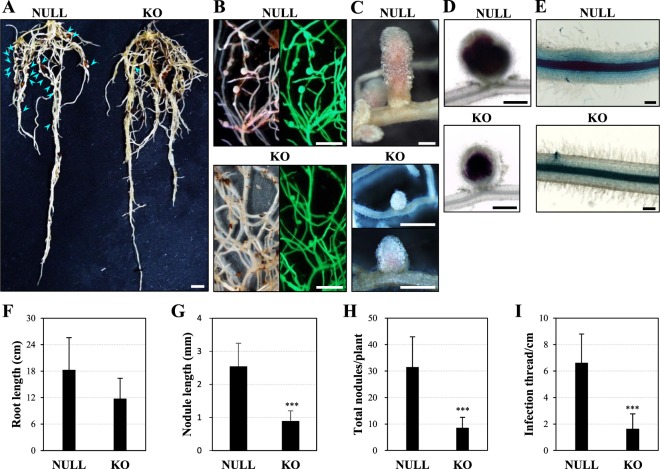
Root and nodule development of the *MtGA2ox10* deletion plantlet. (**A,B**) Roots and nodules of the 2-month-old NULL and G851.907 KO plantlets. Arrowheads indicate nodules developed from 4 wpi with *S. medicae*. (**C**) Typical mature nodule of the NULL and the largest nodule of the G851.907 KO plantlets are shown. (**D**) *S. medicae* expressing *LacZ* in the nodules of the NULL and G851.907 KO plantlets stained using Magenta-Gal as a substrate. (**E**) Epidermal infection of *S. medicae* of the NULL and G851.907 KO roots. Scale bars are 1 cm (**A,B**) or 500 µm (**C–E**). (**F–I**) Root length (**F**), nodule length (**G**), nodule number per plantlet (**H**), and infection thread number per cm in the root differentiation zone (**I**) were measured. Error bars depict the standard error calculated from six NULL and seven G851.907 KO plantlets. Asterisks represent statistical significance (^***^p < 0.001) by t-test.

Deletion of *MtGA2ox10* strongly affected both nodule number and development on the transformed roots of *M. truncatula* (Fig. 5). Root growth over 2 months in pots of Perlite showed no significant difference of root length between G851.907 KOs and the control roots transformed with the empty vector (Fig. 5A,F). In contrast, the number and size of the nodules were significantly reduced in the G851.907 KO roots (Fig. 5B,C). Unlike the fully grown, cylindrical pink nodules, which measured ~2.5 mm in length on the control roots, G851.907 KO roots formed pale, white, immature nodules that measured <1 mm in length (p < 0.001; Fig. 5G) and were on average 3.7-fold fewer in number (p < 0.001; Fig. 5H). Interestingly, there were no significant differences in rhizobial colonization or zonal organization of similar-sized nodules between G851.907 KOs and the control, as seen by staining of LacZ activity (Fig. 5D). On the other hand, the number of infection thread per cm in the differentiation zone of G851.907 KO roots was 4.0-fold fewer than in the control (p < 0.001), indicating that epidermal infection of rhizobia was highly affected in G851.907 KO roots (Fig. 5E,H).

### Over-expression of *MtGA2ox10* causes a dwarf phenotype and inhibition of nodule formation

**Figure 6:**
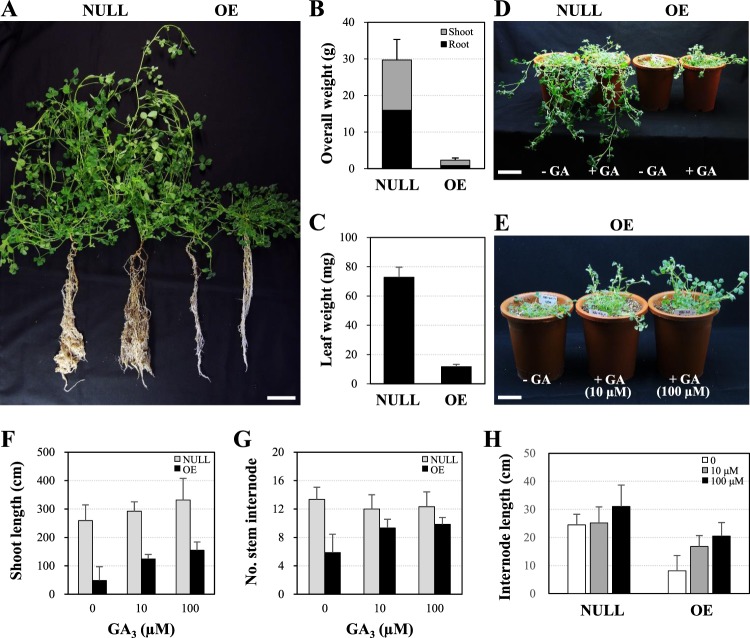
Effect of *MtGA2ox10* over-expression on plant architecture and growth of the stable transgenic plantlet. (**A**) Photograph of the 2-month-old NULL and *MtGA2ox10* OE stable transgenic plantlets. (**B,C**) Fresh weight of overall plant (**B**) or leaf (**C**) of the transgenic plantlet. (**D**) Effect of GA_3_ application on the growth phenotype of the transgenic plantlets. Photograph was taken 2 weeks after exogenous application of 10 µM GA_3_ on the transgenic plants. (**E**) Dose-dependent growth phenotype of the *MtGA2ox10* OE stable transgenic plantlets. Photograph was taken 2 weeks after exogenous application of 10 µM or 100 µM GA_3_ on the transgenic plants. (**F–H**) Shoot length (**F**), number of stem internode (**G**), and internode length (**H**) per plantlet were measured. Error bars depict the standard error calculated from nine NULL and nine *MtGA2ox10* OE plantlets. Scale bars are 5 cm (**A,D,E**).

To assess the effect of ectopic expression of *MtGA2ox10* in plant growth and nodule development, *MtGA2ox10* was over-expressed under the *CaMV 35S* promoter in the *A. tumefaciens*-transformed stable transgenic plants. A total of 16 independent stable transgenic plants were selected and analyzed. As shown in Fig. 6, over-expression of *MtGA2ox10* (*MtGA2ox10* OE) affected plant architecture. Two-month-old transgenic plants grown in pots showed characteristics of GA-deficient phenotypes; dwarfism, small dark-green leaves, and reduced stem and root growth. Biomass of the *MtGA2ox10* OE plants was only 7.8% to that of the control plants (Fig. 6A–C). Moreover, all of the T_0_ plants of *MtGA2ox10* OE failed to yield seeds even with application of GA_3_. *MtGA2ox10* OE in *A. rhizogenes*-transformed hairy roots also showed a ~1.8-fold decreased root mass compared to the control (Supplementary Fig. S4). To test whether exogenous application of GA could rescue the dwarf phenotypes of the *MtGA2ox10* OE stable transgenic plants, nine independent transgenic plants were treated with GA_3_ at concentrations of 10 µM or 100 µM through irrigation. GA_3_ application resulted in a dose-dependent recovery of plant growth in two weeks after the application (Fig. 6D,E). The transgenic plants showed different sensitivity of growth response to GA_3_ compared with the control lines. Changes in the number of stem internode and length of stem internode were obvious in the *MtGA2ox10* OE lines but not in the control lines at 10 µM GA_3_ (Fig. 6F to H).

**Figure 7:**
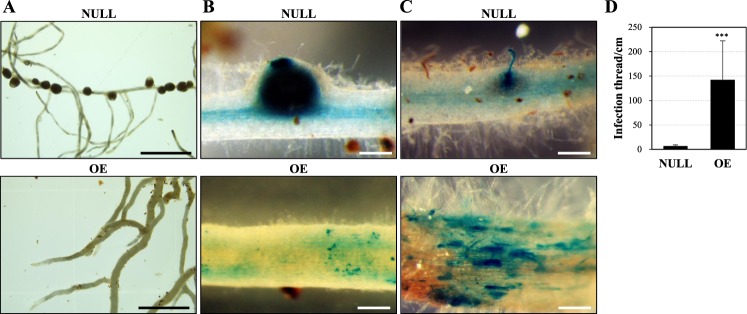
Nodule development of the *MtGA2ox10* over-expression stable transgenic plantlet. (**A**) Roots of the 2-month-old NULL and *MtGA2ox10* OE stable transgenic plantlets at 3 wpi with *S. medicae*. (**B**) *S. medicae* expressing *LacZ* in the nodules of the NULL and on the root epidermis of the *MtGA2ox10* OE stable transgenic plantlets at 3 wpi with *S. medicae*. (**C**) Epidermal infection of *S. medicae* of the NULL and *MtGA2ox10* OE roots at 4 days post inoculation with *S. medicae*. Scale bars are 5 mm (**A**) or 200 µm (**B,C**). (**D**) Infection thread number per cm in the root differentiation zone was measured. Error bars depict the standard error calculated from four NULL and six *MtGA2ox10* OE plantlets. Asterisks represent statistical significance (^***^p < 0.001) by t-test.

qPCR analysis of GA metabolic pathway genes in the *MtGA2ox10* OE transgenic plants displayed more than a 2-fold increased expression of *ent*-kaurene synthesis-related genes (*KS* in root and *KAO* in leaf) and GA oxidase genes (*CYP714A1* and *GA3ox* in root and *CYP714C1* in leaf) (Supplementary Fig. S5). This result showed that the over-expression of *MtGA2ox10* differentially altered the relative transcript levels of GA synthesis pathway genes in root and leaf of transgenic plants compared with the control lines. *MtGA2ox10* OE also significantly affected nodulation (Fig. 7). In the control lines (n = 4), a number of nodules formed at 3 weeks post inoculation of *S. medicae* ABS7M (Fig. 7A). In contrast, lines over-expressing *MtGA2ox10* had 23-fold increase in the number of infection threads compared with the control line (p < 0.001). However, no nodules were detected on the roots of *MtGA2ox10* OE stable transgenic plants (n = 6) even after 4 weeks post rhizobium inoculation (Fig. 7B to D). Meanwhile, approximately a 1.9-fold fewer nodules formed per *A. rhizogenes*-transformed plant; however, no prominent difference of nodule structure or rhizobial colonization was observed in the mature nodule (Supplementary Fig. S4).

## Discussion

Symbiotic nodule organogenesis is a complex developmental reprograming process that requires tight regulation of the interaction between the rhizobium and the host plant. Plant hormones are important positive or negative regulators of legume-rhizobial symbiosis, as they affect the expression of symbiotic genes. Larrainzar *et al*.^27^ noted that symbiosis-specific transcriptional activation of biosynthetic pathways for multiple plant hormones, such as ethylene, cytokinin, abscisic acid, GA, and strigolactone, takes place within hours of inoculation with the rhizobium, suggesting that these hormones likely interact to regulate downstream symbiotic responses. Interestingly, this study also reported on nuanced aspects of the GA anabolic and catabolic pathways. Both GA biosynthesis and inactivation pathway genes were upregulated, with temporal differences in a Nod factor-dependent manner. Consistent with previous suggestions^3–5^, our findings provide new insights into the activity of GA during nodulation and show that spatiotemporal regulation of GA in nodule development must be considered not only in biosynthesis, but also in catabolism.

Previous studies of the roles of GA in nodulation have focused on the GA biosynthesis gene or the DELLA-mediated downstream signaling pathway. A low GA concentration is essential for the initial stage of infection, but inhibits the normal progress of nodule organogenesis. Therefore, GA levels must be regulated dynamically and differentially during the separate stages of nodulation, epidermal infection and nodule organogenesis^4,5^. In contrast, little attention has focused on the inactivation or transport of GA compared to biosynthesis and signaling in nodulation. In this study, we characterized the molecular function of *MtGA2ox10* encoding the C20 GA-specific inactivation enzyme GA2-oxidase in symbiotic nodule organogenesis. This novel *MtGA2ox* gene exhibited rhizobium-dependent induction in the 6 to 36 hpi window, and negative regulation by ethylene in the *M. truncatula* root. Gene expression was induced as early as at 6 hpi and peaked at 12 hpi in wild type A17; it was highly enhanced in *skl* but was markedly low in *nfp* and *lyk3*. Native promoter::GUS fusion analysis confirmed that transcriptional activation of the *MtGA2ox10* promoter was associated with rhizobium infection and nodule development. The formation of infection thread, as well as the number and size of nodules, were reduced by CRISPR/Cas9-mediated deletion of *MtGA2ox10*. Additionally, plant architecture and nodulation were also affected by over-expression of *MtGA2ox10*, whereas exogenous application of GA_3_ rescued the dwarf phenotype. These findings collectively suggested that *MtGA2ox10* is a unique member of the *MtGA2ox* gene family, controlling the low concentration of GA by catabolic inactivation of C20 GA in roots during epidermal infection of the rhizobium. Therefore, it plays as a catabolic regulator of symbiotic nodule organogenesis.

MtGA2ox10 clustered into subgroup III GA2ox with substrate specificity to C20 GA, but not to active C19 GAs (Fig. 1). A number of studies have reported on the significance of C20 GA regulation for plant responses and organ development. Two C20 *GA2ox* genes, *AtGA2ox7* and *AtGA2ox8*, control plant architecture and floral initiation in *A. thaliana*^17,32^. C20 *GA2ox* is also related to tillering and root development^8^, as well as to salt tolerance and root gravity responses^33^ in rice, and over-expression of a C20 *GA2ox* in switchgrass changes the plant architecture, for example through increased tillering, a short internode length, and reduced plant height^34^. It was interesting to note that all of the reported phenotypes of C20 *GA2ox* over-expression showed less severe dwarfism compared to C19 *GA2ox* over-expression, suggesting that C20 GA2ox does not completely deplete the pools of diverse GAs and may have a more specialized role in plant development. Meanwhile, *MtGA2ox10* OE in the stable transgenic plants resulted in dwarfism with low fertility and inhibition of nodule development despite of increased root infection, presumably due to ectopic inactivation of earlier intermediate C20 GAs (GA_12_ and GA_53_) or disruption of the GA pool by altered expression of *KS*, *KAO*, *GA13ox*, and *GA3ox*. These results were consistent with the previous report from pea *na-1* mutant^5^; therefore, clarified the role of GA on the different stages of nodulation (suppression of infection and activation of nodule formation). Of particular interest, the stable transgenic plants of *MtGA2ox10* OE showed different root growth and nodulation pattern compared with the hairy root transformation lines (almost normal development of root and nodule). We anticipate that GAs transported to the *A. rhizogenes*-transformed roots from the aerial parts might compensate for the effect of *MtGA2ox10* OE as demonstrated by grafting experiments in GA-deficient mutant pea and *A. thaliana*^14,35^.

GA biosynthesis is a complex and multistep process with diverse intermediates. Therefore, it is difficult to determine the exact spatial localization of GA biosynthesis. Other studies have suggested that GAs are mobile signaling molecules in plants. The successful completion of a number of development processes requires GAs to be mobile^36^. A study of pea using radiolabeled forms of GA_19_, GA_20_, and GA_1_ showed that GA_20_ was the major mobile form of GA in the pea^35^. In *A. thaliana*, the biologically inactive C20 GA_12_ is the major transported form of GA^13,14^. The membrane permeability of GA_12_ allows it to serve as a long-distance transport molecule^36^. Considering the fact that the *A. rhizogenes*-transformed hairy roots of *MtGA2ox10* OE formed normal nodules and *MtGA2ox10pro::GUS* expression occurred in the vascular bundles of the roots and mature nodules but not near the base of mature nodules, GA transport through the vascular system in *M. truncatula* is expected to be under catabolic regulation by C20 GA-specific *MtGA2ox* and GA precursors are converted to active forms at the location where the nodule develops. Additionally, expression of *MtGA2ox10* in the mature nodule suggests that it may inhibit nodule over-growth by quantitative regulation of GA, which is a known regulator of cell expansion and cell cycle activation. Further analysis such as grafting of wild-type scions onto rootstocks of stable transgenic over-expression and knock out lines or measurement of GA content in the transgenic plants will prove this hypothesis.

In conclusion, this study described the importance of fine catabolic tuning of GA for nodule development in *M. truncatula*. We clarified that *MtGA2ox10* is a unique member of the *MtGA2ox* gene family regulating rhizobium infection and nodule organogenesis. This is the first report on the roles of the GA catabolic pathway gene in nodulation of legume plants and contributes towards a more comprehensive understanding of the dynamic nature of the GA regulatory mechanism. Research is underway to establish and characterize stable transformed plants with loss-of-function for *MtGA2ox*, to further understand the roles of GA and its regulation through catabolism and transport for symbiotic nodule development.

## Methods

### Plant growth conditions and inoculation of rhizobium bacteria

*M. truncatula* cv. Jemalong A17 seeds were scarified, germinated, and grown in a growth room at 22 °C under 16 h light/8 h dark conditions. For rhizobium inoculation of the seedlings, germinated 1-day-old seedlings were planted on the aeroponic caisson, a large plastic chamber with a perforated lid on top and a humidifier that sits on the bottom^37^, where they were misted with Lullien’s aeroponic culture medium^38^ containing 0.5 mM ammonium nitrate. The 2-week-old seedlings were inoculated with *S. medicae* ABS7M (pXLGD4) constitutively expressing the *LacZ* gene at an optical density at 600 nm (OD600) of 0.1. For rhizobium inoculation of *A. rhizogenes*-mediated transformed roots, 4-week-old transformed plantlets were transferred to Perlite in 1 L pots and maintained for 2 weeks with a supplement of half strength modified Fahraeus medium (mFM) containing 0.5 mM ammonium nitrate. Six-week-old transformed plantlets were then inoculated with *S. medicae* ABS7M (pXLGD4) at OD600 of 0.05.

### Phylogenetic analysis of the *GA2-oxidase* gene family

For phylogenetic analysis of the *GA2ox* gene family in the sequenced plant genomes, putative *GA2ox* genes in the genomes of *B. rapa*, *G. max*, *L. japonicus*, *M. truncatula*, *O. sativa*, *S. lycopersicon*, and *V. vinifera*, were identified based on a BLASTP search (E value cutoff of E^−10^ and query coverage of 50%) using *A. thaliana GA2ox* genes as the seed queries. At the same time, the GA2ox protein sequences of tomato^31^, rice^8^, and grapevine^39^ were downloaded from the National Center for Biotechnology Information (NCBI) GenBank database and combined with the BLASTP search results. The deduced amino acid sequences of the *GA2ox* genes were aligned using the ClustalW program^40^ with the default parameters. The phylogenetic tree was constructed using the Maximum-Likelihood method in MEGA7^41^, with bootstrap analysis of 1,000 replicates for stability testing of the tree nodes. Identification of other GA biosynthesis pathway genes, including *CPS*, *KS*, *KO*, *KAO*, *GA13OX*, and *GA3ox*, in the *M. truncatula* genome (Mt4.0) was also performed by BLASTP search (E value cutoff of E^−10^ and query coverage of 50%) using the previously reported GA biosynthesis genes of *M. truncatula*^42^ as the seed queries.

### Transcriptional expression analyses

For the transcriptome analysis, our RNA-seq data, which were deposited to NCBI under the BioProject accession number PRJNA269201, were mapped to the very recent *M. truncatula* genome assembly Mt4.0, as described previously^27^. Read counts were normalized using the trimmed mean of M-values (TMM) method^43^. Average TMM values for the GA metabolic pathway genes per sample were selected and analyzed by hierarchical clustering using Cluster 3^44^. A heat map was drawn with the log-transformed fold changes of the TMM values compared to 0 hpi of A17 as a control. For the qPCR analysis of *MtGA2ox10*, plant roots were harvested at 0, 6, 12, 24, 48 hpi and 2 weeks post-inoculation (wpi) with *S. medicae* ABS7M. Leaves and flowers were sampled from 8-week-old plants. Un-inoculated roots from 4-week-old plants were included as a control. Total RNA was extracted using the CTAB method^45^ combined with LiCl precipitation and DNase treatment using the TURBO DNA-free kit (Ambion, Life Technologies, Carlsbad, CA, USA). First strand cDNA was synthesized using the TOPscript^TM^ cDNA synthesis kit (Enzynomics, Daejeon, Korea) with oligo-dT. The cDNAs were diluted 10-fold and qPCR was performed using TOPreal^TM^ qPCR premix (Enzynomics) and a CFX96™ Real-Time PCR Detection System (Bio-Rad, Hercules, CA, USA). The comparative cycle threshold (Ct) method, also known as the 2^−DDCt^ method^46^, was employed for relative quantification using the *GAPDH* gene (Medtr3g085850) as a reference gene. qPCR analysis of other GA biosynthesis pathway genes (*CPS*, *KS*, *KO*, *KAO*, *GA13ox*, and *GA3ox*) was also performed using the oligonucleotide primers designed to amplify target genes from the closely related family genes (Supplementary Table S3).

### Gene cloning and plasmid construction

All of the primers used in plasmid construction are listed in Supplementary Table S4. To construct the promoter::GUS reporter fusion, 2.1 kb upstream of the 5′-flanking region of the *MtGA2ox10* gene (Medtr4g074130) was amplified from the genomic DNA of *M. truncatula* A17 using Phusion High Fidelity DNA polymerase (Thermo Fisher Scientific, Waltham, MA, USA). The resulting PCR amplicon was purified by agarose gel electrophoresis and cloned into pDONR221 using BP Clonase II (Thermo Fisher Scientific). The binary destination vector pRNGWFS7 (Kim, unpublished) was constructed by replacing *NPT II* in pKGWFS7^47^ with the *DsRed*::*NPT II* translational fusion under the *CaMV 35S* promoter. The entry plasmid was recombined with pRNGWFS7 in the presence of LR Clonase II (Thermo Fisher Scientific) to obtain a transcriptional fusion of the *MtGA2ox10* promoter to GFP and GUS (*MtGA2ox10pro::GUS*). To construct the over-expression vector, the full-length coding sequence (CDS) of *MtGA2ox10* was amplified from the first strand cDNA which was synthesized with the total RNA isolated from the *S. medicae*-infected root tissues of *M. truncatula* A17. The amplicon was cloned into pDONR221 using BP Clonase II (Thermo Fisher Scientific) and recombined with pK7WG2D^47^ using LR Clonase II (Thermo Fisher Scientific) to obtain the binary construct for over-expression of the *MtGA2ox10* CDS under the *CaMV 35S* promoter. The binary Cas9 expression vector pGK3304 and the sgRNA cloning vector pGK2223 were constructed as follows; the Cas9 expression cassette consisting of the *CaMV 35S* promoter, *Cas9::NLS::HA* and the *CaMV 35S* terminator was PCR-amplified from pBAtC^48^. A *Hin*d III site within *Cas9* was removed by overlap PCR and the Cas9 expression cassette was transferred to pKGWD^47^ by replacing the GFP expression cassette between the *Sac* I and *Hin*d III sites. The resulting plasmid was named pKGWC. A fragment of the *CaMV 35S* promoter, *GFP(S64T)::BAR*, and the *NOS* terminator were amplified from pGK2720 (Kim, unpublished) and replaced the Kanamycin resistant gene in pKGWC to yield pGK3304. A sgRNA cloning vector was constructed by placing the gRNA cloning site and gRNA scaffold in pBAtC^48^ under the promoter of the *MtU6–8* small nuclear RNA gene promoter in pENTR_MtU6.8::gus0::UT^30^. The resulting Gateway-compatible sgRNA cloning vector was named pGK2206 and the *Aar* I cloning site in pGK2206 was replaced by the *Bsa* I cloning site to yield pGK2223.

### CRISPR/Cas9-mediated deletion

For the CRISPR/Cas9-mediated deletion of *MtGA2ox10*, two sgRNAs were designed on exon 3 of *MtGA2ox10* gene using Cas-Designer^49^. The complementary oligonucleotides were annealed and cloned into the *Bsa* I cloning site of the entry vector pGK2223 using the Golden Gate assembly method^50^. Briefly, two complementary oligonucleotides were phosphorylated using T4 polynucleotide kinase (NEB, Ipswich, MA, USA) and annealed in a kinase buffer. The annealed oligonucleotides were mixed with pGK2223 plasmid, *Bsa* I and T4 DNA ligase (NEB). The reaction mixture was incubated at 37 °C for 30 min, and then subjected to 30 cycles of 5 min at 37 °C and 10 min at 24 °C. After a final incubation at 50 °C for 30 min, the Golden Gate assembly was transformed into *E. coli* TOP10 cells. Two entry plasmids with different sgRNA were tandem assembled using the restriction cloning method. One sgRNA expression cassette was cut out from the entry plasmid using *Xba* I and *Spe* I and inserted into another sgRNA entry plasmid which was digested by *Xba* I and dephosphorylated. The resulting dual sgRNA entry plasmid was recombined with the binary CRISPR/Cas9 vector pGK3304 using the LR clonase II (Thermo Fisher Scientific).

### Plant transformation

For *A. rhizogenes*-mediated hairy root transformation, the binary constructs were electroporated into *A. rhizogenes* MSU440 and transformed roots were generated in *M. truncatula* A17 as previously described^51^. To select the plantlets, glufosinate herbicide BASTA^TM^ (Bayer Crop Science, Monheim am Rhein, Germany) was added to the medium at a concentration of 4 mg/l and the growing hairy roots were selected by detection of GFP using an IZX2-ILLB stereomicroscope equipped with a GFP filter set (Olympus, Tokyo, Japan). One transformed root was left for each plantlet while all non-transformed roots were removed. Four-week-old composite plantlets with transformed roots were transferred to Perlite in a 1 L pot and grown in a growth room as described above. For *A. tumefaciens*-mediated stable transformation, the binary constructs were electroporated into *A. tumefaciens* EHA105 and stable transgenic plants of *M. truncatula* A17 was generated as previously described^52^. Briefly, sterilized leaf explants of *M. truncatula* A17 were co-cultivated with *A. tumefaciens* on the P4 medium and callus was induced on the P4 medium containing 5 µM GA_3_ (Sigma-Aldrich, https://www.sigmaaldrich.com), 40 mg/L Kanamycin (Sigma-Aldrich), and 400 mg/L Cefotaxime (Sigma Aldrich). The transgenic somatic embryos were removed from the callus tissue and were plated onto the MS medium containing 10 g/L sucrose, 50 mg/L Kanamycin, and 0.25% Gelrite for development into plantlets. When sufficiently grown, plantlets were transferred to Perlite in a 1 L pot and grown in a growth room as described above.

### Histochemical staining and fluorometric quantification of *LacZ* and *GUS* expression

Plant roots were harvested at 6, 12, 24, 48 hpi and 2 wpi with *S. medicae* ABS7M. Transformed roots were selected by detecting GFP under a fluorescence stereomicroscope as described above. The constitutive expression of *LacZ* in *S. medicae* ABS7M was detected using X-Gal as a substrate according to a standard protocol^53^. Dual staining of LacZ and GUS was carried out according to the protocol in the *L. japonicus* handbook^54^. The reaction was monitored overnight to avoid over-staining. Fluorometric quantification of GUS activity was conducted using 4-methylumbelliferyl b-D-glucuronide as a substrate^55^. The fluorescence was measured with a DynaQaunt 200^TM^ fluorometer (Hoeffer, San Francisco, CA, USA).

### Genotyping by PCR-RFLP and sequencing

Genomic DNA was extracted from the transformed hairy roots of the *A. rhizogenes*-transformed composite plantlets or leaves of the stable transgenic plants using the standard CTAB method^56^ for PCR, cloning, and sequencing. In parallel, a simple boiling method in 25 mM NaOH for genotyping by RFLP was applied. The CRISPR/Cas9-targeted region of *MtGA2ox10* was amplified with the 2289-F and 2905-R primers, using Phusion High Fidelity DNA polymerase (Thermo Fisher Scientific). The amplicons were digested using the *Bsr*D I (Thermo Fisher Scientific) or *Eco105* I (Enzynomics) restriction enzymes and analyzed by agarose gel electrophoresis. Additionally, the amplicon was sequenced using the 2347-F primer after being cloned in the pLPS-TOPO Blunt vector (Elpis Biotech, Daejeon, Korea). Genotyping of the stable transgenic plants was performed by PCR amplification of the *MtGA2ox10* coding sequence in the binary plasmid using G512-F and P35S-SF primers.

### GA treatment and statistics test

GA_3_ (Sigma-Aldrich) was dissolved in ethanol at stock concentration of 10 mM. Two-month-old stable transgenic plants grown in pots were supplemented with nitrogen-free mFM medium containing either of 10 µM or 100 µM GA_3_ at final concentration. Changes in plant architecture were recorded for four weeks. To statistically test the difference in measurements, the independent t-test was performed using SPSS.

## Supplementary information

Supplementary Information

## Data Availability

The RNA-seq data used in this study have been deposited in NCBI’s Bioproject collection under the Bioproject ID PRJNA269201.
