# Supplementary material for: MtGA2ox10 encoding C20-GA2-oxidase regulates rhizobial infection and nodule development in Medicago truncatula
Source: Sci Rep. 2019 Apr 11;9:5952. doi: 10.1038/s41598-019-42407-3 (PMC6459840; doi:10.1038/s41598-019-42407-3)
Supplement: Supplementary file 1 — Supplementary Information [file 41598_2019_42407_MOESM1_ESM.pdf]

MtGA2ox10 encoding C20-GA2-oxidase regulates rhizobial infection and nodule development in *Medicago truncatula*

Goon-Bo Kim[1], Seong-Uk Son[1], Hee-Ju Yu[2], Jeong-Hwan Mun[1,*]

[1]Department of Bioscience and Bioinformatics, Myongji University, Yongin 17058, Korea

[2]Department of Life Science, The Catholic University of Korea, Bucheon 14662, Korea

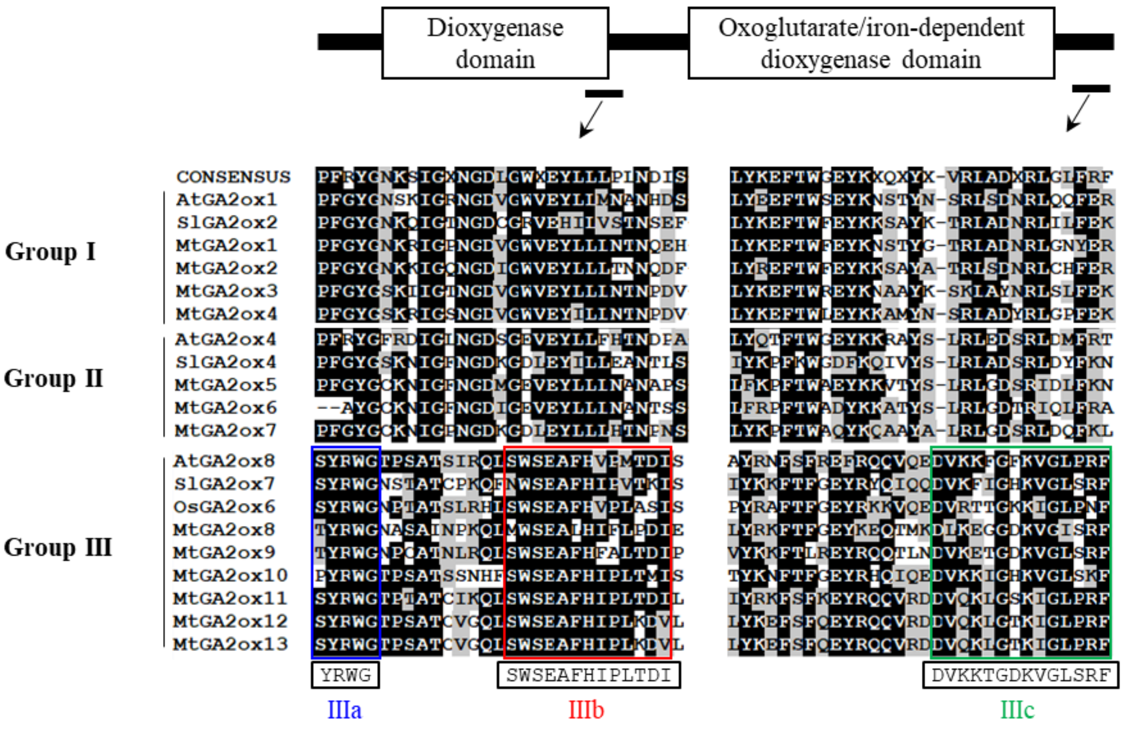

Figure S1. Multiple alignments of MtGA2ox proteins with their homologs of *A. thaliana* (At), *S. lycopersicon* (Sl), and *O. sativa* (Os). Alignments of dioxygenase domain and C-terminal regions are presented. Group I and II contain C19 GA2ox whereas Group III comprises of C20 GA2ox. Three amino acid motifs (IIIa, IIIb, and IIIc) conserved in C20 GA2ox are indicated in color boxes.

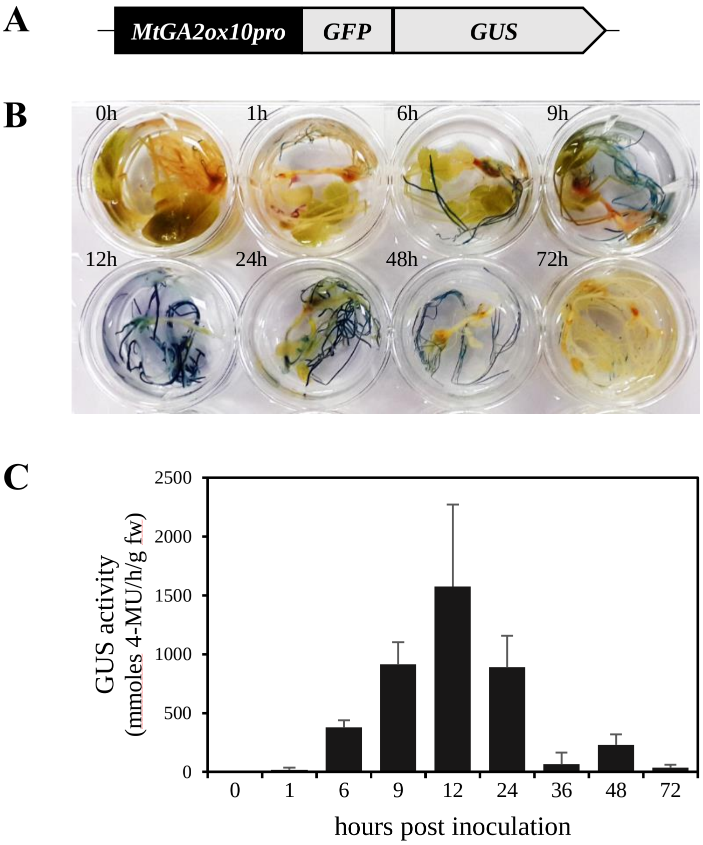

Figure S2. Promoter activity of *MtGA2ox10*. A, The 2.1 kb 5'-upstream region of *MtGA2ox10* was fused to the *GFP:GUS* reporter gene in pRNGWFS7 (*MtGA2ox10pro:GUS*) and analyzed for promoter::GUS activity in *A. rhizogenes*-transformed roots. B, Four-week-old transformed roots were inoculated with *S. medicae* ABS7M (pXLGD4) and stained for GUS activity using X-Gluc as a substrate. C, GUS activity in the *A. rhizogenes*-transformed roots was quantified using 4-MUG as a substrate. Error bars depict the standard error calculated from nine plantlets at each time point.

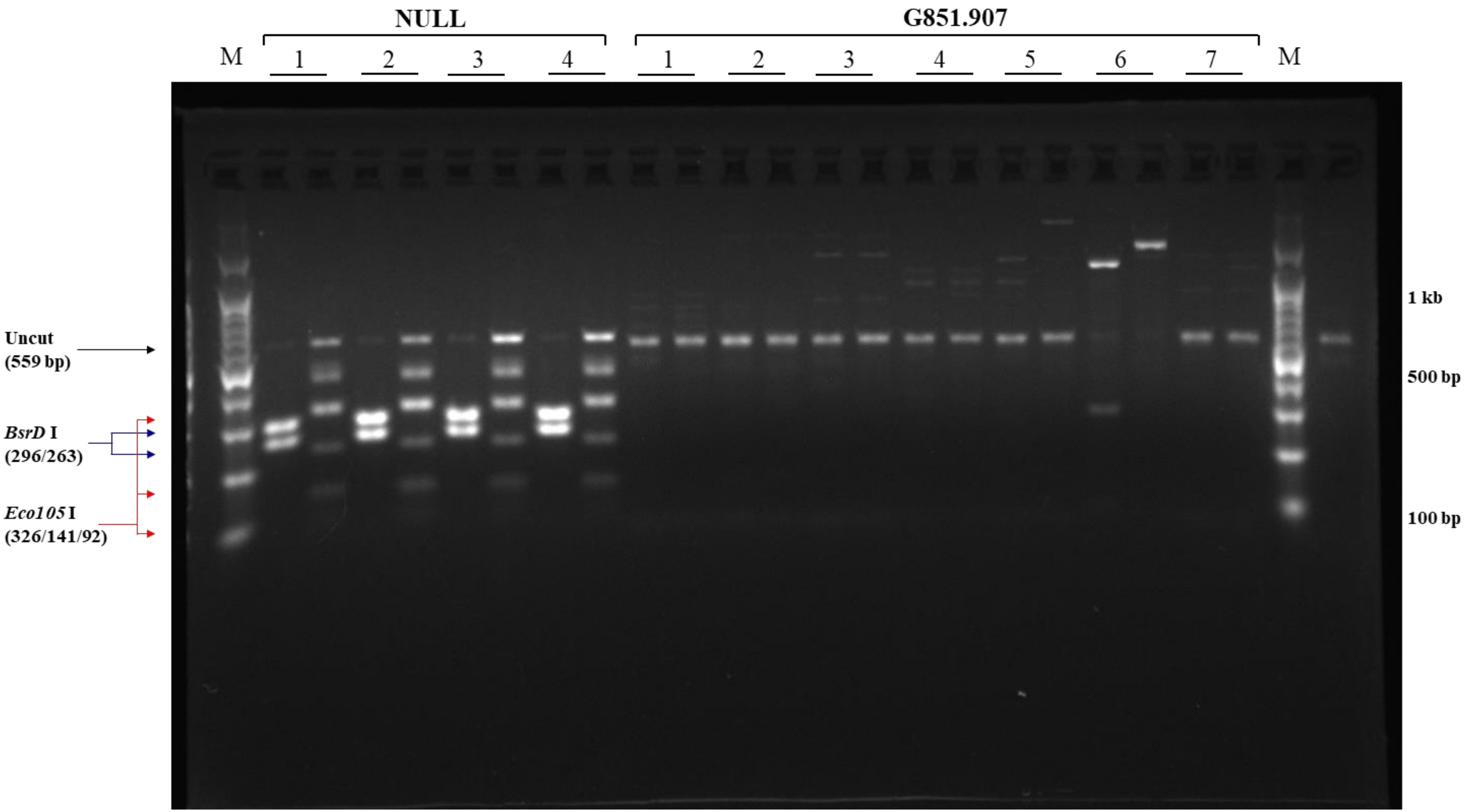

Figure S3. Full-length gel image of Fig.4D representing PCR-RFLP genotyping of *A. rhizogenes*-transformed roots harboring G851.907. PCR amplicons from four root samples of the pGK3304 empty vector (NULL) and seven root samples of G851.907 (G851.907) were digested independently by *BsrD* I (left lane) or *Eco105* I (right lane).

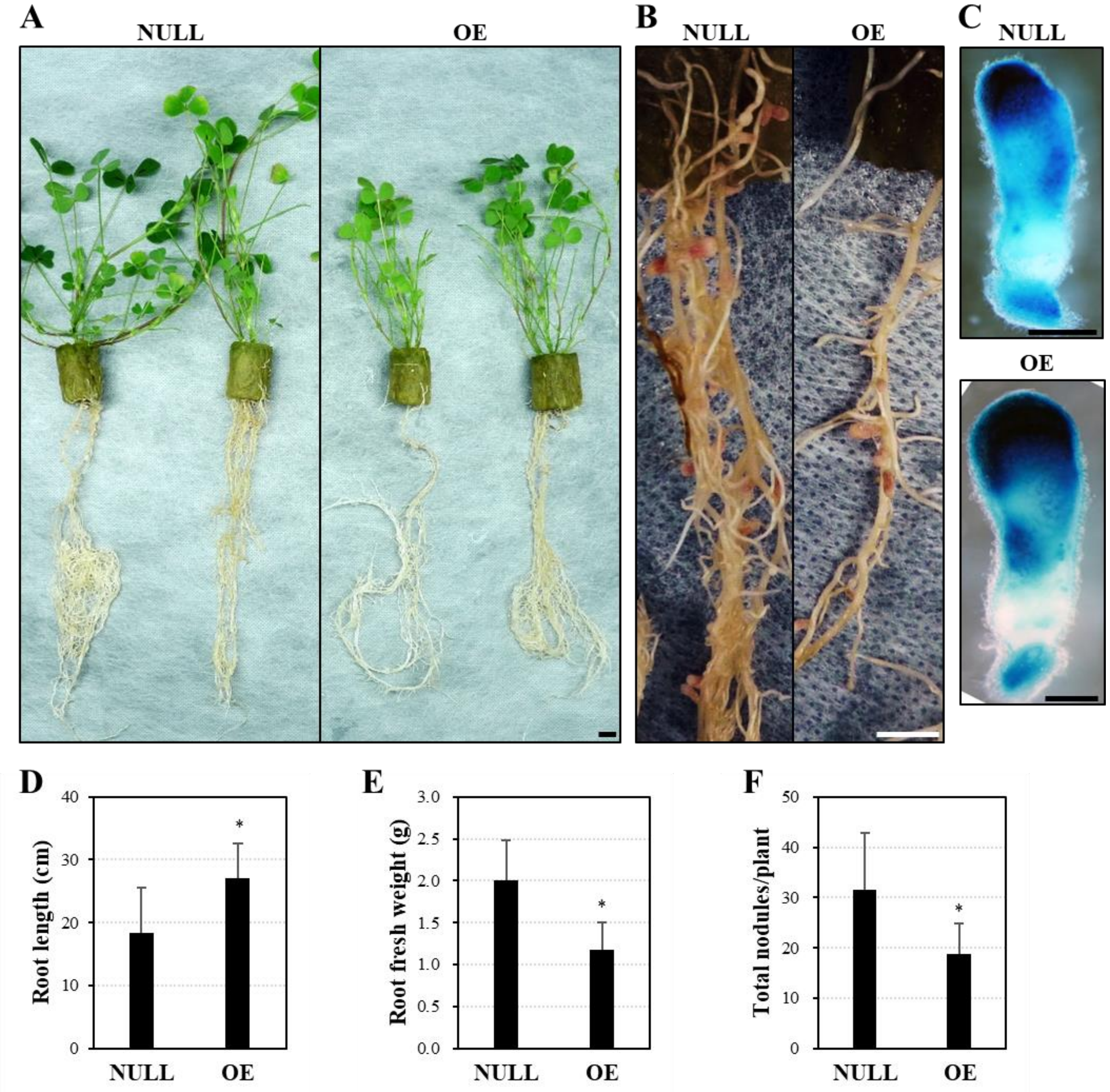

Figure S4. Root and nodule development of the *MtGA2ox10* over-expressing plantlet transformed by *A. rhizogenes*. A, Roots of the 2-month-old NULL and *MtGA2ox10* OE plantlets. B and C, Nodules of the NULL and *MtGA2ox10* OE plantlets developed from 4 wpi with *S. medicae*. *S. medicae* expressing *LacZ* in the nodules of the NULL and *MtGA2ox10* OE plantlets was stained using X-Gal as a substrate (C). Scale bars are 1 cm (A), 5 mm (B), or 500 μm (C). D to F, Root length (D), root fresh weight (E), and nodule number per plantlet (F) were measured. Error bars depict the standard error calculated from six NULL and six *MtGA2ox10* OE plantlets. Asterisks represent statistical significance (* $p < 0.05$) by t-test.

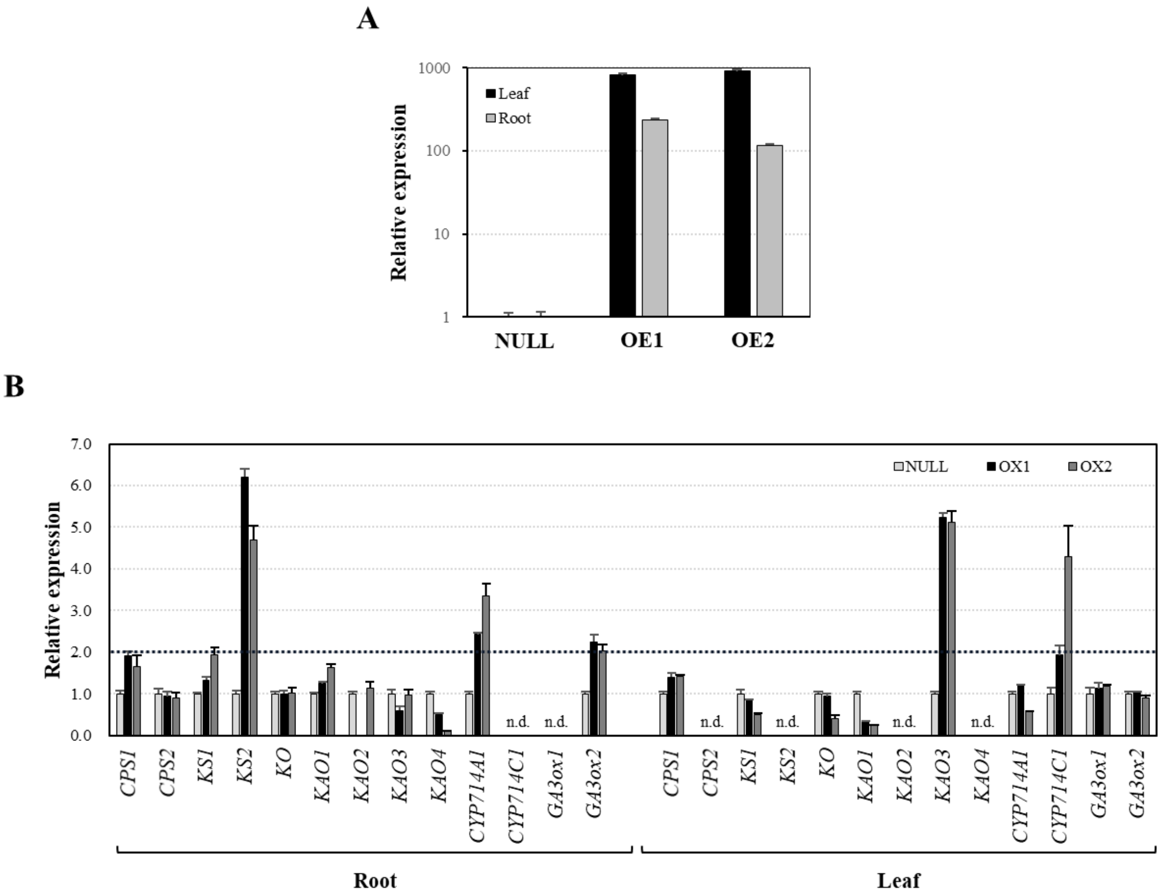

Figure S5. Expression of *MtGA2ox10* (A) as well as other GA biosynthesis-related genes (B) in the stable *MtGA2ox10* OE transgenic lines. Expression level of each gene in root and leaf was verified using qPCR. Values represent the relative expression calculated using the NULL as a reference. Error bars depict the standard error calculated from three independent replicates for each sample.

Table S1. Identification of *GA2ox* genes in *M. truncatula*

At		GA family	Mt ortholog		
Gene ID (TAIR10)	At gene name*	specificity	No.	Gene ID (Mt4.0)	Mt gene name
AT1G78440.1	*GA2ox1*	C19	2	Medtr2g070870.1	*MtGA2ox1*
				Medtr8g461330.1	*MtGA2ox2*
AT1G30040.1	*GA2ox2*	C19	2	Medtr4g096840.1	*MtGA2ox3*
				Medtr2g019370.1	*MtGA2ox4*
AT2G34555.1	*GA2ox3*	C19		no hit	
AT1G47990.1	*GA2ox4*	C19	1	Medtr2g033270.1	*MtGA2ox5*
AT1G02400.1	*GA2ox6*	C19	2	Medtr4g123020.1	*MtGA2ox6*
				Medtr1g086550.1	*MtGA2ox7*
AT1G50960.1	*GA2ox7*	C20		no hit	
AT4G21200.1	*GA2ox8*	C20	6	Medtr2g083000.1	*MtGA2ox8*
				Medtr2g083030.1	*MtGA2ox9*
				Medtr4g074130.1	*MtGA2ox10*
				Medtr5g005570.1	*MtGA2ox11*
				Medtr7g451860.1	*MtGA2ox12*
				Medtr7g047670.1	*MtGA2ox13*
AT5G58660.1	AT5G58660.1	both	1	Medtr3g464530.1	*MtGAOL15*
AT3G47190.1	AT3G47190.1	both		no hit	

**A. thaliana* genes were identified from the gibberellin inactivation I (2β-hydroxylation) in METACyc database.
At, *A. thaliana*; Mt, *M. truncatula*.

Table S2. Normalized TMM values of RNA-seq read counts for GA biosynthesis and inactivation pathway genes of *M. truncatula*[1)]

Genotype[2)]		A17									*nfp*									*lyk*									*skl*								
hpi[3)]		0h	0.5h	1h	3h	6h	12h	24h	36h	48h	0h	0.5h	1h	3h	6h	12h	24h	36h	48h	0h	0.5h	1h	3h	6h	12h	24h	36h	48h	0h	0.5h	1h	3h	6h	12h	24h	36h	48h
Biosynthesis	Medtr1g034120 (GAOL2)	1995	899	880	1202	1703	2336	1337	2010	986	2563	1433	1545	1473	1916	3069	2265	3113	2237	2429	1662	1408	1429	2216	2409	1893	1950	1376	2803	2049	1328	1326	1668	2301	1195	1531	565
	Medtr1g034060 (GAOL1)	1450	823	567	715	1182	1648	911	1407	703	1899	1087	975	912	1474	2326	1636	2150	1565	1812	1112	863	902	1673	2096	1383	1371	931	2087	1462	916	886	1400	1781	869	1037	390
	Medtr2g102570 (GA3ox1)	198	126	133	182	257	426	617	304	289	221	126	193	205	157	145	339	303	444	235	142	119	170	311	402	410	356	318	331	222	241	297	440	651	1110	1140	1267
	Medtr1g011580 (GA3ox2)	164	64	117	211	152	206	223	169	214	192	146	186	192	242	297	311	328	344	270	134	242	272	210	259	299	275	270	187	115	201	230	188	164	146	100	90
	Medtr5g025090 (GAOL13)	140	161	151	159	160	158	162	164	158	143	170	166	142	165	151	155	162	186	145	171	157	155	163	183	158	171	170	152	178	161	163	163	160	146	170	162
	Medtr1g102070 (GA20ox3)	140	88	74	114	81	121	143	114	123	172	139	172	208	206	255	233	222	229	225	186	192	217	170	170	176	143	144	194	148	152	236	163	134	105	79	66
	Medtr4g087140 (GAOL9)	110	95	76	104	96	142	132	138	87	105	115	91	100	127	147	119	139	102	110	93	101	124	113	147	158	139	131	135	127	136	136	138	149	162	153	114
	Medtr4g087100 (GAOL5)	39	97	90	37	37	36	39	30	47	68	71	60	28	31	40	42	37	45	32	58	45	34	34	29	41	38	43	92	110	123	74	74	63	64	56	98
	Medtr3g096500 (GA20ox5)	36	25	30	48	49	48	53	46	32	37	18	26	40	49	34	43	30	41	76	21	13	27	39	30	34	30	40	30	34	30	54	83	61	65	76	98
	Medtr4g087120 (GAOL7)	14	17	18	4	10	21	12	13	12	17	16	12	10	8	13	13	15	15	18	10	13	11	8	23	8	19	14	11	8	6	4	6	15	6	7	6
	Medtr1g081840 (GA20ox2)	3	3	5	4	6	1	5	4	16	7	16	16	7	6	5	14	1	17	15	27	68	30	15	3	21	15	24	8	3	17	11	12	2	9	1	5
	Medtr8g033380 (GA20ox6)	12	11	6	5	5	16	9	7	4	10	5	5	6	4	10	8	13	10	14	10	10	4	6	18	9	9	7	5	5	3	6	3	5	4	6	8
	Medtr1g034140 (GAOL3)	16	1	3	1	2	5	1	2	2	21	2	3	2	2	2	2	4	2	23	2	2	2	3	7	2	5	3	12	1	1	1	1	1	1	1	1
	Medtr4g087110 (GAOL6)	12	2	2	1	3	8	4	5	4	27	2	6	4	2	5	7	6	8	28	4	2	3	4	9	6	8	11	15	4	5	4	2	8	6	5	5
	Medtr4g087090 (GAOL4)	9	5	7	4	4	9	5	8	9	15	9	14	6	11	20	12	20	15	13	5	5	6	6	10	5	13	8	16	11	7	9	7	12	7	8	5
	Medtr6g464620 (GA20ox1)	6	5	4	8	3	3	5	4	6	5	5	2	6	4	4	5	5	4	6	9	3	7	5	6	5	6	7	9	4	3	5	9	6	5	3	1
	Medtr4g087130 (GAOL8)	4	4	2	3	4	5	7	8	5	5	5	5	3	6	6	6	4	5	4	2	3	3	5	4	7	6	6	4	3	5	4	5	4	6	5	3
	Medtr3g088745 (GA20ox4)	0	0	0	0	0	1	3	3	3	0	0	0	0	0	0	0	0	0	0	0	0	0	0	0	0	0	0	0	0	0	0	1	1	6	11	28
	Medtr5g025050 (GAOL11)	1	1	0	1	1	1	1	1	0	9	3	3	4	3	5	3	4	4	3	2	1	2	2	1	1	1	1	2	1	1	1	1	1	0	1	1
Inactivation	Medtr1g086550 (GA2ox7)	193	350	297	277	322	268	201	224	211	221	371	360	320	318	269	195	223	199	229	357	309	253	278	248	184	215	207	176	300	260	250	261	233	133	132	78
	Medtr3g464530 (GAOL15)	65	74	100	130	111	131	166	89	93	60	97	90	141	141	130	114	120	125	78	76	111	113	129	119	133	99	117	74	92	111	128	128	169	236	258	211
	Medtr4g074130 (GA2ox10)	2	3	7	11	56	106	47	28	25	3	6	3	2	2	9	12	19	16	2	7	9	7	29	42	29	33	24	4	5	8	9	82	139	119	96	99
	Medtr4g096840 (GA2ox3)	102	55	54	88	69	82	65	102	85	67	56	45	67	85	87	62	82	53	107	76	85	80	92	86	67	113	89	73	73	64	78	48	46	22	39	22
	Medtr4g123020 (GA2ox6)	16	31	27	10	8	10	13	12	16	19	23	24	15	15	12	26	16	24	18	26	20	11	16	9	19	9	19	23	39	37	20	25	19	16	12	20
	Medtr2g070870 (GA2ox1)	16	12	12	10	17	12	16	16	14	40	14	25	11	10	8	24	13	18	39	16	11	8	16	11	12	8	13	18	10	10	7	9	5	10	7	8
	Medtr2g019370 (GA2ox4)	7	10	14	12	11	13	6	16	8	11	12	14	9	11	12	9	11	8	7	9	11	8	9	12	6	10	4	10	13	13	12	18	15	8	9	5
	Medtr8g461330 (GA2ox2)	0	1	1	1	1	4	2	1	1	2	1	1	0	1	1	1	1	1	3	2	1	1	2	0	0	1	0	1	2	2	3	6	4	3	1	1
	Medtr7g047670 (GA2ox13)	1	1	1	0	0	2	1	1	1	0	0	0	1	1	0	0	1	1	1	0	1	1	0	1	0	1	1	1	1	1	0	2	0	1	0	0
	Medtr2g033270 (GA2ox5)	1	1	1	0	0	1	1	1	2	1	2	1	0	1	1	0	0	1	1	1	2	1	1	0	0	0	0	1	1	1	1	1	1	1	1	1
	Medtr5g005570 (GA2ox11)	1	0	1	1	0	1	1	0	1	0	0	1	1	0	1	1	1	1	0	1	0	1	1	1	1	2	1	1	1	0	1	1	2	3	2	3

[1)]Average TMM values of genes for each time point were quantified based on our previously reported transcriptome data (NCBI BioProject accession number PRJNA269201).

[2)]A17, wild type *M. truncatula* cv. Jemalong A17; *nfp*, loss-of-function mutant of NOD FACTOR PERCEPTION (*NFP*); *lyk*, loss-of-function mutant of LysM domain receptor-like kinases (*LYK3*); *skl*, loss-of-function mutant of ethylene-insensitive (*EIN2*) sickle.

[3)]Hours-post-inoculation with *S. medicae* ABS7M.

Table S3. List of oligonucleotides used in qPCR analysis

Protein family	Gene symbol	Locus (Mt v4)	Sequence (5' to 3')	Aplicon size (bp)	Alternative gene name*
Copalyldiphosphate synthase (CPS)	*CPS1*	Medtr7g011663	AAATTTGCCTGATGAGGTGG<br>TGTTCACATTCGCCATCCTA	149	*CPS*
	CPS2	Medtr7g011770	GAACCTGCCGGGAGAGATAG<br>TGCTCACATTCAACATCCTG	149	
Ent-kaurene synthase (KS)	*KS1*	Medtr2g064295	GTTGCTCCGAGGCAGTTAAGA<br>ATTGCGTCCTTGCCGCTTGA	92	*KS*
	KS2	Medtr3g058160	GGCGAAAAATGGGGTACTCAC<br>GACATCTACATCCCACTTCTCC	112	*KS-like*
Ent-kaurene oxidase (KO)	*KO*	Medtr2g105360	AAAATCGTCAGGACCGTCTGT<br>TCATGGAATACAGCCCCCAAG	109	*KO*
Ent-kaurenoic acid oxidase (KAO)	*KAO1*	Medtr2g031930	TAAGGAAATCCGCGGCATGG<br>GCCTCTCGAAAGACCACCAA	90	*KAO1*
	KAO2	Medtr2g031920	CCACTTTCTCCTCAACTATGAGTTG<br>AGAACGTTTCTTGATCCTTGCC	112	*KAO2*
	KAO3	Medtr5g014240	TGGACCGTTATCAACCTCAC<br>TTGATCGGAAGGCCTTCTTG	93	*Beta-amyrin oxidase1*
	KAO4	Medtr5g014250	CTCGCCATCAATCTCACTGGA<br>TTGATCCACCAAGGCTTGCA	90	
	KAO5	Medtr8g089800	TCTGTCCTGGAGCCGACTTA<br>TGGGTTTATTCGCTCCAACCTG	87	*Beta-amyrin oxidase2*
	KAO6	Medtr8g012970	CGAGAGGCAACAACTGATGC<br>AGCCATATGCATAGCGTTTAGC	90	*Beta-amyrin oxidase3*
Cytochrome P450 (GA13ox)	*CYP714A1*	Medtr3g093530	AGGTTGCTCAACATTGCCCCA<br>AAGGCTGCTGGTGGGTATAACC	112	*CYP714A1*
	CYP714A2	Medtr2g061200	GAGACCTTTGGTAATCTGCCT<br>AGGCCCATAAATACGCATACTC	105	*CYP714A2*
	CYP714C1	Medtr7g055793	CTTGAAGTATGCGGAAATGGT<br>TCTTGGAATGATGTTCTGGTGA	131	*CYP714C1*
	CYP714C2	Medtr8g008530	AACGCCAAATTGATGCTCATG<br>TCTCTTGAAGTGGCGTTTGGT	112	*CYP714C2*
Gibberellin 3-beta-dioxygenase (GA3ox)	*GA3ox1*	Medtr2g102570	GCCAAACACTGTGATACTGTC<br>GAGAGTCCAACATTAGCCACA	85	*GA3ox1*
	GA3ox2	Medtr1g011580	GACTACAAACGGTTTTGTGACA<br>CATGGTTGTTAGAACCAATCCA	139	*GA3ox2*
Gibberellin 2-beta-dioxygenase (GA2ox)	*GA2ox10*	Medtr4g074130	AGCAGCTTGTTGGGGTGAAT<br>ATGTGCTGGCATCACAGAGT	155	
Glyceraldehyde 3-phosphate dehydrogenase	*GAPDH*	Medtr4g103920	TGCTCATTTGAAGGGTGGTGC<br>GATAACCTTTGCAAGTGGTGC	166	

Table S4. List of oligonucleotides used in plasmid construction and genotyping

Name	Sequence (5’ to 3’)	Purpose
AtU6-gRNA-f	gctcGAATTCGAATGATTAGGC	Amplification of gRNA cloning cassette from pBAtC
AtU6-gRNA-r	gagagaattcAAAAAAAAGCACCGACTCGGTGCCAC	
MtU6-8f	taggTCTAGAGGTGCTATTGAAGCTATT	Cloning of MtU6-8 promoter
MtU6-8r	aacaCTCGAGacacctgcctccAATCCTTACGGTTCGCTT	
*Bsa*I- sense	gattAGAGACCTTCTCGAGAGTGGTCTCA	*Bsa* I cassette for guide RNA cloning
*Bsa*I- antisense	aaacTGAGACCACTCTCGAGAaGGTCTCT	
Cas9H3-f	GAGCCGCAAACTTATCAACGGCATCCGCGA	Removal of *Hind* III site in Cas9
Cas9H3-r	CGTTGATAAGttTGCGGCTCAGGCGGC	
2C7-f	tgatatcccgcggatggtgagCGACTAGAGCCAAGCTGAT	Amplification of the Cas9 expression cassette from pBAtC vector
2C7-r	TGATCCAAGCTCAAGCTAAGC	
2FB6-f	TCGATCTAGAGGGCGTATTGGCTAGAGCA	Amplification of GFP-BAR expression cassette from pGK2720
2FB6-r	CGAACTAGTAACATAGATGACACCGCG	
GA2ox10pro-b1	AAAAAGCAGGCTtcAGATTATTGCTCACGGCCTCT	Gateway cloning of *MtGA2ox10* promoter
GA2ox10pro-b2	AGAAAGCTGGGTcTGTAGCACCTAGCTGTTGTGT	
GA2ox10-b1	AAAAAGCAGGCTtcATGATTGACTCAAATCCACC	Gateway cloning of *MtGA2ox10* CDS
GA2ox10-b2	AGAAAGCTGGGTcCTAAGCCATGGTCGTTGTGT	
G851s	gattGAAGGACATAGGAAGTATGCAA	Guide RNA for *MtGA2ox10*, sense
G851as	aaacTTGCATACTTCCTATGTCCTTC	Guide RNA for *MtGA2ox10*, antisense
G907s	gattGTGAAATTCTTATACGTAGA	Guide RNA for *MtGA2ox10*, sense
G907as	aaacTCTACGTATAAGAATTTCAC	Guide RNA for *MtGA2ox10*, antisense
2347-F	ATGATCAAAGTGACTGCAGC	PCR genotyping of CRISPR/Cas9-mediated deletion in *MtGA2ox10* gene
2905-R	ACTCTTGCATATAATCCGAGTGAC	
G512-F	GGACATAGGAAGTATGCAATGG	PCR genotyping of transgenic plants over-expressing *MtGA2ox10* under CaMV 35S promoter
P35S-SF	AGAAGACGTTCCAACCACGT	
